# Association of Sugary Beverage Consumption With Mortality Risk in US Adults: A Secondary Analysis of Data From the REGARDS Study

**DOI:** 10.1001/jamanetworkopen.2019.3121

**Published:** 2019-05-17

**Authors:** Lindsay J. Collin, Suzanne Judd, Monika Safford, Viola Vaccarino, Jean A. Welsh

**Affiliations:** 1 Department of Epidemiology, Emory University, Atlanta, Georgia; 2 Department of Biostatistics, University of Alabama, Birmingham; 3 Department of Medicine, Weill Cornell Medical College, Cornell University, New York, New York; 4 Department of Pediatrics, Emory University, Atlanta, Georgia; 5 Wellness Department, Children’s Healthcare of Atlanta, Atlanta, Georgia

## Abstract

**Importance:**

Research has linked sugar-sweetened beverage (SSB) consumption to coronary heart disease (CHD) risk, but the role of nutritionally similar fruit juice and the association of these beverages with mortality risk is unknown.

**Objective:**

To assess the association of SSBs and 100% fruit juices, alone and in combination (sugary beverages), with mortality.

**Design, Setting, and Participants:**

This cohort study is a secondary analysis of data obtained from 30 183 participants in the Reasons for Geographic and Racial Differences in Stroke (REGARDS) study. The REGARDS study was designed to examine modifiers of stroke risk. Enrollment took place from February 2003 to October 2007, with follow-up every 6 months through 2013. Overall, 30 183 non-Hispanic black and white adults 45 years and older were enrolled in the REGARDS study. Those with known CHD, stroke, or diabetes at baseline (12 253 [40.6%]) and those lacking dietary data (4490 [14.9%]) were excluded from the current study, resulting in a sample size of 13 440. Data were analyzed from November 2017 to December 2018.

**Exposures:**

Sugar-sweetened beverage and 100% fruit juice consumption was estimated using a validated food frequency questionnaire and examined using categories of consumption that align with recommended limits for added sugar intake as a percentage of total energy (TE; <5%, 5%-<10%, and ≥10%) and 12-oz serving increments.

**Main Outcomes and Measures:**

All-cause and CHD-specific mortality were determined from cause of death records and family interviews and adjudicated by a trained team. Multivariable adjusted hazard ratios (HRs) were estimated using regression models.

**Results:**

Overall, 13 440 participants had a mean (SD) age of 63.6 (9.1) years at baseline, 7972 (59.3%) were men, 9266 (68.9%) were non-Hispanic white, and 9482 (70.8%) had overweight or obesity. There were 1000 all-cause and 168 CHD-related deaths during follow-up (mean [SD] follow-up, 6.0 [1.8] years). Mean (SD) sugary beverage consumption was 8.4% (8.3%) of TE/d (4.4% [6.8%] TE/d from SSBs; 4.0% [6.8%] TE/d from 100% fruit juice). Among high (≥10% of TE) vs low (<5% of TE) sugary beverage consumers, risk-adjusted HRs were 1.44 (95% CI, 0.97-2.15) for CHD mortality and 1.14 (95% CI, 0.97-1.33) for all-cause mortality. Risk-adjusted all-cause mortality HRs were 1.11 (95% CI, 1.03-1.19) for each additional 12 oz of sugary beverage consumed and 1.24 (95% CI, 1.09-1.42) for each additional 12 oz of fruit juice consumed. In risk-adjusted models, there was no significant association of sugary beverage consumption with CHD mortality.

**Conclusions and Relevance:**

These findings suggest that consumption of sugary beverages, including fruit juices, is associated with all-cause mortality. Well-powered and longer-term studies are needed to inform their association with CHD mortality risk.

## Key Points

**Question:**

Is the consumption of sugary beverages (ie, sugar-sweetened beverages and fruit juices) associated with an increased mortality risk?

**Findings:**

In this cohort study of 13 440 black and white adults 45 years and older observed for a mean of 6.0 years, each additional 12-oz serving/d of sugary beverages was associated with an 11% higher all-cause mortality risk, and each additional 12-oz serving/d of fruit juice was associated with a 24% higher all-cause mortality risk. Similar associations were not observed for sugary beverage consumption and coronary heart disease mortality.

**Meaning:**

These results suggest higher consumption of sugary beverages, including fruit juice, is associated with increased mortality.

## Teaser

This cohort study and secondary analysis of the Reasons for Geographic and Racial Differences in Stroke (REGARDS) study assesses the association of sugary beverage consumption with mortality among US adults.

## Introduction

Currently, an estimated 1 of 6 deaths in the United States is attributed to coronary heart disease (CHD).^1^ Men, people with low income, and non-Hispanic individuals are among those at highest risk.^2^ In multiple experimental and long-term prospective studies, high consumption of dietary sugars, sugar-sweetened beverages (SSBs) in particular, has been associated with several CHD risk factors, including dyslipidemia,^3^ diabetes,^4^ and obesity,^4,5^ but there has been little research to understand if this risk extends to an increase in associated mortality. Yang et al,^6^ using data from the National Health and Nutrition Examination follow-up survey, demonstrated an increase in cardiovascular disease mortality with higher added sugar intake among US adults, but to our knowledge, no one has yet examined the extent to which mortality risk is elevated with sugary beverages, including SSBs and fruit juices.

The nutrient content of 100% fruit juices and SSBs is very similar. While 100% fruit juices contain some vitamins and phytonutrients that are missing from most SSBs, the predominant ingredients in both are sugar and water.^7^ Although the sugar in SSBs is added during processing and the sugar in 100% fruit juice occurs naturally, the specific sugars they provide for the body to process are essentially the same,^8^ and the biochemical response when metabolized is the same. The sugars contained in all sugary beverages are primarily the monosaccharides glucose and fructose or the disaccharide sucrose, which is quickly broken down with digestion and metabolized into equal parts fructose and glucose.

In recent years, public health efforts to promote a reduction in the consumption of SSBs and other sources of added sugars in the United States have intensified,^9,10,11^ but despite some declines, consumption remains well above recommended levels.^12^ Less attention has been given to the role of 100% fruit juice consumption, which tends to be perceived as a healthy beverage option. Therefore, to inform policy and the development of dietary guidelines, it is critical to understand how beverages high in naturally occurring sugars, in addition to those high in added sugars, are associated with cardiovascular health and mortality risk. The purpose of this study is to examine the association of higher consumption of sugary beverages, alone and in combination, with mortality risk.

## Methods

### Study Population

The Reasons for Geographic and Racial Differences in Stroke (REGARDS) study is a large US-based cohort study of adults 45 years and older at baseline, designed to identify factors that contribute to the excess stroke mortality among people living in the southeastern United States and among black US residents.^13^ Details of the REGARDS cohort and its sample design have been described in detail elsewhere.^13^ Briefly, participants were randomly selected using commercially available lists of US residents with the goal of recruiting half of the study sample from the stroke buckle (coastal North and South Carolina and some parts of Georgia) and the stroke belt (remaining areas of North Carolina, South Carolina, and Georgia as well as Tennessee, Mississippi, Alabama, Louisiana, and Arkansas) and the other half from the rest of the continental United States.^13^ Within each region, the goal was to recruit half non-Hispanic white and half non-Hispanic black participants as well as half male and half female participants. Individuals were recruited through mail and telephone between February 2003 and October 2007. Follow-up data collected through 2013 were used in this analysis, which began in November 2017 and was completed in December 2018. The REGARDS longitudinal cohort has had an overall attrition rate of 24.7% (7381 participants).

To minimize the risk of detecting an association resulting from reverse causality owing to dietary changes following a disease diagnosis associated with increased CHD mortality risk, we excluded those with a self-reported history of CHD (5886 [19.5%]), type 2 diabetes (4920 [16.3%]), and stroke or transient ischemic attack (1447 [46.0%]) at baseline as well as those missing dietary data (4490 [14.9%]) from the total enrolled in the REGARDS study (30 183), yielding a final sample at baseline of 13 440 individuals (49.5% of total REGARDS sample).

Written informed consent was obtained from all participants, and the study was approved by the institutional review boards at all participating institutions. This cohort study followed the Strengthening the Reporting of Observational Studies in Epidemiology (STROBE) reporting guideline.^14^

### Exposure Assessment

On enrollment in the REGARDS study, diet was assessed using a self-administered Block 98 food frequency questionnaire (FFQ), a validated semiquantitative FFQ that assesses the usual dietary consumption of 110 food items (NutritionQuest).^15,16^ For each food item included in the FFQ, participants were asked about their usual consumption patterns during the preceding year, with response options ranging from never to every day. In addition to frequency of consumption, participants were asked to estimate the usual quantity of food consumed as either the number of specified units or the portion of food served on a plate. The FFQ survey form was given to participants during the baseline in-home visit. Once completed, they were mailed by participants in preaddressed envelopes to the REGARDS operations center. Questionnaires were verified for completeness and sent to NutritionQuest for analysis.

Sugary beverages were defined as SSBs (ie, sodas, soft drinks, or fruit-flavored drinks) and naturally sweet 100% fruit juices. After FFQ processing, an estimate of the daily consumption of each of these beverages was provided in grams for each participant. This amount was summed to estimate each individual’s total consumption of sugary beverages. Calorie information available on the US Department of Agriculture Food Composition Database was used to estimate the calories per gram for each sugary beverage type.^7^ To estimate the contribution of each to total energy (TE), beverage calories were divided by TE consumption for each participant. Consumption was then grouped using cutoffs that align with the upper limits for sugar intake advised by US Dietary Guidelines, the World Health Organization, and the American Heart Association.^10^ These groupings were low (<5%), medium (5%-<10%), and high (≥10%). Given the common usage of ounces in the United States, grams were converted to ounces by dividing by 31.^7^ Further analysis was done to assess the linear association by examining the association with each additional 12-oz serving.

### Outcome Assessment

The outcomes of interest were CHD-related and all-cause mortality. Study participants (or their family members) were interviewed by telephone every 6 months to log all hospital visits or death events.^17^ In addition, some death events were identified when family members or friends called the REGARDS study’s toll-free numbers to report them. Patients who died in the hospital had their cause of death recorded in their medical records; for those who died outside of a hospital setting, interviews with family members, death certificates, and the National Death Index were used to identify date and cause of death. Other death events were identified through searches of the Social Security Administration Master Death File. Adjudication was then done by clinicians (general internists, cardiologists, and physician assistants) who had undergone specific training to identify causes of death. This group reviewed dates and causes of death by examining death certificates, medical records, and other administrative databases.^17^ Analyses in the present study were based on preliminary or verified dates of death as of December 31, 2013. The REGARDS longitudinal cohort has had an overall attrition rate of 24.7% (7381 participants). However, previous studies examining the association of attrition with reported estimates found minimal bias.^18^

### Covariates

Following enrollment and informed consent, REGARDS staff completed a telephone interview to collect information on medical history, personal history, physical activity, depressive symptoms, cognitive function, quality of life, and social support. Following the telephone interview, REGARDS staff scheduled a baseline in-home visit to take anthropometric measurements and collect blood and urine samples. Participants were also given a self-administered FFQ to complete and mail back to research staff.

Covariates were chosen based on a priori knowledge of factors associated with sugary beverage consumption and CHD mortality risk, including age (continuous in years), sex (male or female), education (some college or less), household income (<$74 000 or ≥$74 000), region (stroke belt, stroke buckle, or other), smoking (current, former, or never), alcohol consumption (none, moderate, or heavy), physical activity (none, 1-3, or ≥4 times per week), body mass index (continuous), fiber consumption (grams), saturated fat consumption (grams), and TE consumption (continuous).

### Statistical Analysis

To describe the sample, we calculated the proportion of participants in each of several demographic, anthropometric, and lifestyle subgroups for the full sample and by sugary beverage consumption level as a percentage of TE intake (low, <5%; medium, 5%-<10%; high, ≥10%). We then estimated the mean and median consumption of SSBs and 100% fruit juice separately and in combination (as a percentage of TE as well as in grams and ounces) for each subgroup.

We used multivariable adjusted Cox proportional hazard regression to estimate the hazard of CHD-specific and all-cause mortality associated with increasing consumption of these beverages, first by grouping participants by their level of sugar intake and then on an individual basis with each additional 12 oz consumed. Years since study entry was the time metric, with participants censored by date of death, date of withdrawal from the study, or December 31, 2013, whichever came first. We verified the proportional hazards assumption for the exposure variables and potential confounders using natural log–natural log survival curves and with the inclusion of an interaction term of the covariate with time.

Statistical analysis began with an unadjusted model. Demographic variables, smoking, and alcohol use were added in model 2, body mass index was added in model 3, and physical activity and diet were added in model 4. As we were unable to control for income owing to missing data from a large proportion of participants (1557 [11.6%]), we did a sensitivity analysis among the subsample for whom income data were available. Given the possible role of TE as a mediator in the association of sugary beverage consumption with mortality risk, the results from the final model with and without TE consumption were examined.

We evaluated the presence of multiplicative interaction between each additional 12-oz serving of sugary beverages using model 4, although with key variables dichotomized as needed to facilitate interaction testing and interpretation of results. The dichotomized versions of variables used in these models were education (<high school vs ≥college) and body mass index status (overweight and obese vs underweight and normal). Testing was done using a likelihood ratio test. We used *P* < .05 as an indicator of statistical significance for all analyses, and tests were 2-tailed. All analyses were performed using SAS version 9.4 (SAS Institute).

## Results

A description of the demographic, anthropometric, and lifestyle characteristics of all the 13 440 participants (representing 79 442 person-years) and of participants categorized by their sugary beverage consumption level is provided in Table 1. The mean (SD) age of the sample was 63.6 (9.1) years at baseline, and most were male (7972 [59.3%]), white (9266 [68.9%]), and had overweight or obesity (9482 [70.8%]). Those who were excluded from the study because they did not have dietary data (4490 [33.4%]) were more likely to be female, be non-Hispanic black, have overweight or obesity, be former or current smokers, and have less than a high school education (eTable 1 in the Supplement). Nearly all of the study population (13 091 individuals [97.4%]) reported consuming some sugary beverages; 10 873 individuals (80.9%) reported consuming SSBs, and 12 637 individuals (94.0%) reported consuming fruit juices (Table 2). Estimated mean (SD) daily consumption was 317 (334) g or 8.4% (8.3%) TE for all sugary beverages, 4.4% (6.8%) TE for SSBs, and 4.0% (6.8%) TE for 100% fruit juice.

**Table 1.** Characteristics of 13 440 US Adults in the REGARDS Study by Sugary Beverage Consumption

Characteristic	No. (%)			
	All Participants	Sugary Beverage Intake		
		0%-<5% of TE	5%-<10% of TE	≥10% of TE
Total	13 440 (100)	5686 (42.3)	3493 (26.0)	4261 (31.7)
Age, y				
45-54	1937 (14.4)	896 (46.3)	423 (21.8)	618 (31.9)
55-64	5625 (41.9)	2415 (42.9)	1411 (25.1)	1799 (32.0)
≥65	5878 (43.7)	2375 (40.4)	1659 (28.2)	1844 (31.3)
Sex				
Male	7972 (59.3)	2053 (37.6)	1593 (29.1)	1822 (33.3)
Female	5468 (40.7)	3633 (45.6)	1900 (23.8)	2439 (30.6)
Race				
White	9266 (68.9)	4568 (49.3)	2454 (26.5)	2244 (24.2)
Black	4174 (31.1)	1118 (26.8)	1039 (24.9)	2017 (48.3)
Education				
<High school	954 (7.1)	335 (35.1)	230 (24.1)	389 (40.8)
High school	3265 (24.3)	1345 (41.2)	772 (23.6)	1148 (35.2)
Some college	3696 (27.5)	1520 (41.1)	959 (26.0)	1217 (32.9)
≥College	5519 (41.1)	2483 (45.0)	1531 (27.7)	1505 (35.3)
Missing	6	3	1	2
Income, $				
<20 000	1708 (12.7)	575 (33.7)	402 (23.5)	731 (42.8)
20 000-<35 000	3051 (22.7)	1188 (38.9)	796 (26.1)	1067 (35.0)
35 000-<75 000	4417 (32.9)	1911 (43.3)	1183 (26.8)	1323 (30.0)
≥75 000	2707 (20.1)	1303 (48.1)	715 (26.4)	689 (25.5)
Missing	1557 (11.6)	709 (45.5)	397 (25.5)	451 (29.0)
Region				
Stroke belt	4579 (34.1)	1908 (41.7)	1146 (25.0)	1525 (33.3)
Stroke buckle	2904 (21.6)	1258 (43.3)	729 (25.1)	917 (31.6)
Other	5957 (44.3)	2520 (42.3)	1618 (27.2)	1819 (30.5)
Weight status^a^				
Underweight	152 (1.1)	63 (41.5)	39 (25.7)	50 (32.9)
Normal	3744 (28.0)	1694 (45.3)	988 (26.4)	1062 (28.4)
Overweight	5182 (38.7)	2191 (42.3)	1376 (26.6)	1615 (35.1)
Obese	4300 (32.1)	1711 (39.8)	1079 (25.1)	1510 (35.1)
Missing	62	27	11	24
Dietary consumption, mean (SD), g				
TE	1726 (709.3)	1705 (707.5)	1812 (692.1)	1685 (719.8)
Total fiber	16.1 (8.5)	17.1 (9.0)	17.3 (8.5)	13.8 (7.3)
Total saturated fat	20.8 (10.7)	21.9 (11.1)	21.8 (10.4)	18.6 (10.2)
Physical activity, times/wk				
0	3997 (30.1)	1622 (40.6)	1015 (25.4)	1360 (34.0)
1-3	5113 (38.6)	2215 (43.3)	1322 (25.9)	1576 (30.8)
>4	4151 (31.3)	1773 (42.7)	1117 (26.9)	1261 (30.4)
Missing	179	76	39	64
Alcohol consumption				
None	7396 (55.9)	2858 (38.6)	1866 (25.2)	2672 (36.1)
Moderate	5144 (38.9)	2379 (46.3)	1416 (27.5)	1349 (26.2)
Heavy	684 (5.2)	372 (54.4)	152 (22.2)	160 (23.4)
Missing	216	77	59	80
Smoking				
Current	1805 (13.5)	706 (39.1)	407 (22.6)	692 (38.3)
Former	5136 (38.4)	2301 (44.8)	1363 (26.5)	1472 (28.7)
Never	6448 (48.2)	2662 (41.3)	1706 (26.5)	2080 (32.3)
Missing	51	17	17	17

Abbreviations: REGARDS, Reasons for Geographic and Racial Differences in Stroke; TE, total energy.

^a^ Weight status categorized as follows: underweight (body mass index [BMI; calculated as weight in kilograms divided by height in meters squared], <18.5), normal (BMI, 18.5-24.9), overweight (BMI, 25.0-29.9), and obese (BMI ≥30).

**Table 2.** Sugary Beverage Intake Among 13 440 Adults 45 Years and Older in the REGARDS Study

Type of Beverage	Prevalence, No. (%)	% TE Intake		Intake, g	
		Mean (SD)	Median (IQR)	Mean (SD)	Median (IQR)
Sugary beverages^a^	13 091 (97.4)	8.4 (8.3)	6.3 (2.1-11.9)	316.5 (334.1)	238.1 (70.5-424.9)
Sugar-sweetened beverages	10 873 (80.9)	4.4 (6.8)	1.3 (0.2-6.1)	173.1 (275.5)	50.5 (6.0-232.2)
100% fruit juices	12 637 (94.0)	4.0 (6.8)	2.3 (0.5-6.0)	143.4 (173.1)	79.4 (16.4-248.8)

Abbreviations: IQR, interquartile range; REGARDS, Reasons for Geographic and Racial Differences in Stroke; TE, total energy.

Conversion factor: To convert grams into ounces, divide by 31.

^a^ Sugar-sweetened beverages, including sodas, soft drinks, fruit drinks, and fruit juice combined.

A total of 168 individuals in the sample died of CHD-related causes, and 1000 died from any cause during the follow-up period (mean [SD] 6.0 [1.8] years) (Table 3). In unadjusted models, consumption of SSBs and 100% fruit juice alone and in combination was positively associated with CHD and all-cause mortality (Figure; Table 3). In model 1, the unadjusted hazard ratio (HR) of CHD mortality when comparing high with low sugary beverage consumers was 2.21 (95% CI, 1.53-3.20) for CHD mortality and 1.31 (95% CI, 1.13-1.51) for all-cause mortality. These results were attenuated with the stepwise addition of covariates, which included demographic characteristics and high-risk practices (race, age at baseline, sex, education, smoking, and alcohol consumption) in model 2, body mass index in model 3, and dietary factors (saturated fat and fiber consumption) and physical activity in model 4 (Table 3). In the fully adjusted model 4, comparing high with low sugary beverage consumers, the adjusted HR of CHD-mortality was 1.44 (95% CI, 0.97-2.15) and of all-cause mortality was 1.14 (95% CI, 0.97-1.33). In our sensitivity analysis, there was little change in the HRs when the CHD mortality analysis was repeated controlling for household income among the subsample with available income data. In that analysis, the adjusted HR comparing high with low sugary beverage consumers was 1.41 (95% CI, 0.93-2.16).

**Table 3.** Risk of CHD and All-Cause Mortality Associated With Intake of Sugary Beverages Among 13 440 Adults in the REGARDS Study

Consumption Level	No. of Cases	HR (95% CI)			
		Model 1^a^	Model 2^a^	Model 3^a^	Model 4^a^
**CHD Mortality**					
0%-<5% TE	45	1 [Reference]	1 [Reference]	1 [Reference]	1 [Reference]
5%-<10% TE	45	1.53 (1.01-2.32)	1.21 (0.79-1.85)	1.21 (0.79-1.85)	1.16 (0.76-1.78)
≥10% TE	78	2.21 (1.53-3.20)	1.66 (1.13-2.43)	1.61 (1.09-2.37)	1.44 (0.97-2.15)
**All-Cause Mortality**					
0%-<5% TE	363	1 [Reference]	1 [Reference]	1 [Reference]	1 [Reference]
5%-<10% TE	258	1.08 (0.92-1.26)	0.91 (0.77-1.08)	0.91 (0.77-1.07)	0.92 (0.78-1.09)
≥10% TE	379	1.31 (1.13-1.51)	1.16 (1.00-1.35)	1.15 (0.99-1.34)	1.14 (0.97-1.33)

Abbreviations: CHD, coronary heart disease; HR, hazard ratio; REGARDS, Reasons for Geographic and Racial Differences in Stroke; TE, total energy intake.

^a^ Model 1 is unadjusted; model 2 adjusted for demographic characteristics, smoking, and alcohol use; model 3 adjusted for demographic characteristics, smoking, alcohol use, and body mass index; and model 4 adjusted for demographic characteristics, smoking, alcohol use, body mass index, physical activity, and diet.

**Figure.**
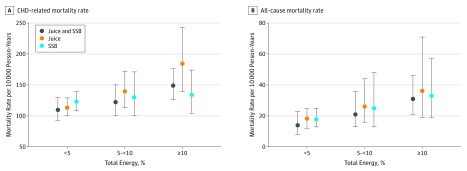
Coronary Heart Disease (CHD)–Specific and All-Cause Mortality Rates Among 13 440 US Adults in the Reasons for Geographic and Racial Differences in Stroke (REGARDS) Study Unadjusted mortality rates at follow-up among US adults in the REGARDS study who consumed 0% to less than 5%, 5% to less than 10%, and 10% or greater of total energy as sugary beverages (ie, 100% fruit juice and sugar-sweetened beverages [SSB]) alone and in combination. Error bars indicate 95% CIs.

As TE is hypothesized to be on the causal pathway between sugary beverage consumption and CHD mortality, it was not included in the main models assessing the association of sugary beverage consumption with mortality, but it was added to model 4 to assess its effect on the estimates. With the addition of TE consumption, estimates were further attenuated, resulting in an HR of 1.31 (95% CI, 0.86-2.00) among the highest vs lowest consumers.

In similarly adjusted models examining sugary beverage consumption as a continuous variable, the HR for CHD mortality with each additional 12-oz serving of sugary beverages was 1.15 (95% CI, 0.97-1.37) and for all-cause mortality was 1.11 (95% CI, 1.03-1.19) (Table 4). We also examined the independent association of SSBs and fruit juices. The HRs of CHD-mortality with each additional 12 oz consumed daily were 1.11 (95% CI, 0.90-1.39) for SSBs and 1.28 (95% CI, 0.95-1.74) for fruit juices. The HRs for all-cause mortality with each additional 12-oz serving were 1.06 (95% CI, 0.96-1.16) for SSBs and 1.24 (95% CI, 1.09-1.42) for fruit juices (Table 4).

**Table 4.** Risk of CHD and All-Cause Mortality Associated With Each Additional 12 Oz of Sugary Beverages Consumed Among 13 440 Adults in the REGARDS Study^a^

Characteristic	CHD Mortality		All-Cause Mortality	
	HR (95% CI)	*P* Value^b^	HR (95% CI)	*P* Value^b^
**Beverage Type**				
All sugary beverages	1.15 (0.97-1.37)	NA	1.11 (1.03-1.19)	NA
SSBs	1.11 (0.90-1.39)	NA	1.06 (0.96-1.16)	NA
Fruit juices	1.28 (0.95-1.74)	NA	1.24 (1.09-1.42)	NA
**All Sugary Beverages **				
Race				
White	1.30 (1.02-1.67)	.20	1.03 (0.93-1.15)	.05
Black	1.05 (0.83-1.32)		1.19 (1.08-1.31)	
Sex				
Male	1.27 (1.01-1.59)	.24	1.20 (1.08-1.33)	.04
Female	1.06 (0.84-1.33)		1.03 (0.93-1.14)	
Education				
≤High school	1.26 (1.03-1.54)	.17	1.20 (1.09-1.33)	.02
≥College	1.01 (0.76-1.32)		1.02 (0.92-1.13)	
Overweight^c^				
No	0.96 (0.69-1.36)	.24	1.01 (0.88-1.16)	.11
Yes	1.21 (1.01-1.46)		1.15 (1.06-1.25)	

Abbreviations: CHD, coronary heart disease; HR, hazard ratio; NA, not applicable; REGARDS, Reasons for Geographic and Racial Differences in Stroke; SSBs, sugar-sweetened beverages.

^a^ All models controlled for potential confounders and modifiers plus age, physical activity, fiber and saturated fat intake, alcohol consumption, and smoking history.

^b^ *P* value for test of interaction between factor and association of sugary beverages with outcome.

^c^ Overweight status defined as body mass index (BMI; calculated as weight in kilograms divided by height in meters squared) of 25.0 or greater.

Our interaction assessment by age, sex, education, or race/ethnicity was done using the models with sugary beverage consumption as a continuous variable. We observed no significant modification by age, sex, education, or race/ethnicity with CHD mortality as the outcome. However, we did observe significant modification in the models with all-cause mortality as the outcome. The interactions were significant by sex and education, with risk highest for men (HR, 1.20; 95% CI, 1.08-1.33; *P* = .04) and those with a high school education or less (HR, 1.20; 95% CI, 1.09-1.33; *P* = .02) (Table 4). With CHD mortality as the outcome, there were no significant interactions by age, sex, education, or race/ethnicity for either SSBs or fruit juice alone. Similarly, there was no evidence of significant modification with fruit juice consumption when all-cause mortality was the outcome (eTable 2 in the Supplement).

## Discussion

The findings of this study suggest that higher consumption of sugary beverages, including fruit juices, among older adults is associated with increased all-cause mortality. These results support those reported previously by Yang et al,^6^ who demonstrated that increasing added sugar intake from foods and beverages increased risk of cardiovascular mortality among US adults, with risk approximately twice that among the highest quintile of consumers compared with the lowest. Our findings extend those obtained in the earlier study by providing evidence specific to sugary beverages only and by demonstrating that risk appears to be elevated with fruit juices alone.

There are a number of possible biological mechanisms to explain an elevated risk of mortality with higher sugary beverage consumption. Obesity, with its link to both SSB consumption and heart disease, may be an important mediator in some cases.^19^ However, given that the higher risk with sugary beverage consumption occurs even when controlling for BMI and the lack of significant interaction between sugary beverage consumption and weight status, our findings suggest that other factors are at play. A 2015 meta-analysis examining the association of SSBs with type 2 diabetes^20^ reported that the incidence of type 2 diabetes rose 18% (95% CI, 9%-28%) and 13% (95% CI, 6%-21%) before and after adjusting for adiposity, respectively, with each additional serving. These results suggest that sugary beverages increase insulin resistance and CHD mortality risk independent of adiposity. Insulin resistance is known to increase triglyceride levels and atherosclerosis, which are important cardiovascular disease risk factors.^21^ Our finding showing an attenuation of the association with the addition of TE consumption to the models suggests that the association of sugary beverage consumption with mortality that we observed may, at least in part, be because of an increase in TE consumption when these beverages are consumed.

The metabolism of fructose, which is unique from all other sugars, occurs unregulated and almost exclusively in the liver. Fructose consumption is known to alter blood lipid levels, markers of inflammation and blood pressure, while high glucose consumption has been associated with insulin resistance and diabetes, independent of weight status.^4,22^ Fructose consumption may also stimulate a hormonal response that promotes fat deposition centrally. Greater central adiposity is a long-recognized cardiovascular disease risk factor. In addition, research suggests that calories consumed in liquid form can increase obesity risk owing to an incomplete compensation for the calories they contain.^23,24^

Given the prominent role that sugary beverages play in the US diet, these results provide support for public health efforts to reduce consumption. Importantly, while an increasing number of program and policy initiatives have focused on reducing the consumption of SSBs, our results suggest that these efforts should be extended to include fruit juices.

### Strengths and Limitations

Our study has many strengths. First, it is based on data collected from a well-defined population-based cohort study with minimal loss to follow-up and well-documented information on important covariates. Second, the REGARDS study population includes a national sample of non-Hispanic black and white adults, which increases the generalizability of our results. Furthermore, data on the complete diet allowed us to assess sugary beverage consumption relative to total dietary consumption. This made it possible to examine how the association with CHD mortality differed when sugary beverage consumption was within vs higher than recommended limits for sugar consumption. We were also able to examine how the risk of CHD mortality changes with each additional serving of sugary beverages (SSBs and fruit juices combined) as well as with each serving of these beverages independently. The use of a validated dietary assessment instrument designed to assess diet patterns over the previous year helped to minimize issues associated with possible seasonal variation in consumption. Also, the availability of adjudicated mortality data reduced the risk of outcome misclassification.

This study is not without limitations. Despite the availability of a large national sample, the number of participants who died during the relatively short follow-up period was small. This increases the risk of a type 2 error, particularly in stratified analyses. In addition, sugary beverage consumption was based on self-report, which is subject to an underreporting bias, specifically for SSBs, that has been shown to differ by a respondent’s weight status, among other factors.^25^ In addition, beverage exposure estimates were available only at baseline. The extent to which that measure reflects consumption throughout the follow-up period is unknown. Furthermore, we were unable to estimate consumption of all types of SSBs, including sweetened teas, which is known to be high among some adults. Nevertheless, it is important to note that the absence of these data is likely to have biased the observed associations toward the null. Third, nearly one-third of the REGARDS cohort did not complete an FFQ, which may have led to selection bias, compromising the interval validity of our study. The REGARDS longitudinal cohort also had an attrition rate of 24.7%. Previous studies examining the association of attrition with reported estimates found minimal bias.^18^ Fifth, we were unable to control for income in the full sample owing to a large amount of missing data. While the results of our subsample analysis suggest that any possible confounding owing to income was addressed by the inclusion of other correlated variables in the model, there may be other unmeasured confounders, such as access to health care, or residual confounding biasing our results.

## Conclusions

In conclusion, the results of this study suggest that higher consumption of sugary beverages, including sugar-sweetened sodas, soft drinks, and fruit drinks as well as naturally sweet fruit juices, is associated with increased all-cause mortality among older US adults. Further well-powered studies with long-term follow-up are needed to clearly delineate the role that sugary beverages play in mortality risk.

## Notes

Supplement. **eTable 1.** Comparison of REGARDS Participants With Dietary Data (and Included in Current Study) and Those Excluded Owing to Lack of Dietary Data **eTable 2.** Association of Each Additional 12 oz of Sugary Beverages Consumed With CHD-Related Mortality Among US Adults >45 Years in the REGARDS Study by Sex, Race, Education, and Weight Status
