# Supplementary material for: Association of Sugary Beverage Consumption With Mortality Risk in US Adults: A Secondary Analysis of Data From the REGARDS Study
Source: JAMA Netw Open. 2019 May 17;2(5):e193121. doi: 10.1001/jamanetworkopen.2019.3121 (PMC6537924; doi:10.1001/jamanetworkopen.2019.3121)
Supplement: Supplement. — eTable 1. Comparison of REGARDS Participants With Dietary Data (and Included in Current Study) and Those Excluded Owing to Lack of Dietary Data eTable 2. Association of Each Additional 12 oz of Sugary Beverages Consumed With CHD-Related Mortality Among US Adults >45 Years in the REGARDS Study by Sex, Race, Education, and Weight Status [file jamanetwopen-2-e193121-s001.pdf]

Supplementary Online Content

Collin LJ, Judd S, Safford M, Vaccarino V, Welsh JA. Association of sugary beverage consumption with mortality risk in US adults: a secondary analysis of data from the REGARDS study. *JAMA Netw Open.* 2019;2(5):e193121. doi:10.1001/jamanetworkopen.2019.3121

eTable 1. Comparison of REGARDS Participants With Dietary Data (and Included in Current Study) and Those Excluded Owing to Lack of Dietary Data

eTable 2. Association of Each Additional 12 oz of Sugary Beverages Consumed With CHD-Related Mortality Among US Adults >45 Years in the REGARDS Study by Sex, Race, Education, and Weight Status

This supplementary material has been provided by the authors to give readers additional information about their work.

© 2019 Collin LJ et al. *JAMA Network Open.*

eTable 1. Comparison of REGARDS Participants With Dietary Data (and Included in Current Study) and Those Excluded Owing to Lack of Dietary Data

	Study Sample (w/FFA)		Excluded (No FFQ)	
	N	%	N	%
**All**	13440	100	4490	100
**Age**				
45-54	1937	14.4	873	19.4
55-64	5625	41.9	1768	39.4
>65	5878	43.7	1849	41.2
**Gender**				
Male	7972	59.3	2010	44.8
Female	5468	40.7	2480	55.2
**Race**				
White	9266	68.9	1857	41.4
Black	4174	31.1	2633	58.6
**Education**				
<High School	954	7.1	694	15.5
High School	3265	24.3	1143	25.5
Some College	3696	27.5	1190	26.5
College or more	5519	41.1	1461	32.6
**Income**				
<$20,000	1708	12.7	893	19.9
$20,000-$35,000	3051	22.7	1027	22.9
$35,000-$75,000	4417	32.9	1280	28.5
>$75,000	2707	20.1	729	16.2
refused	1557	11.6	561	12.5
**Weight status**				
Underweight	152	1.1	53	1.2
Normal	3744	28	1077	24.1
Overweight	5182	38.7	1619	36.2
Obese	4300	32.1	1719	38.5
**Physical activity**				
Inactive	3997	30.1	1505	34.1
1-3 times/week	5113	38.6	1585	35.9
≥4 times/week	4151	31.3	1323	30.0
**Alcohol consumption**				
None	7396	55.9	2860	65.2
Moderate	5144	38.9	1362	31.1
Heavy	684	5.2	163	3.7
**Smoking**				

© 2019 Collin LJ et al. *JAMA Network Open.*

Current	1805	13.5	753	16.9
Former	5136	38.4	2175	48.7
Never	6448	48.2	1542	34.5

© 2019 Collin LJ et al. *JAMA Network Open.*

eTable 2. Association of Each Additional 12 oz of Sugary Beverages Consumed with CHD-Related Mortality among US Adults >45 Years in the REGARDS Study by Sex, Race, Education, and Weight Status[a]

	SSB			100% Juice Only		
	CHD			CHD		
	**HR**	**95% CI**	p-value[b]	**HR**	**95% CI**	p-value[b]
**All**	1.1	0.89–1.38	NA	1.3	0.95–1.74	NA
**Race**						
White	1.2	0.92–1.66	p=0.33	1.5	0.94–2.32	p=0.45
Black	1.0	0.74–1.37		1.2	0.77–1.76	
**Sex**						
Male	1.2	0.86–1.59	p=0.64	1.3	1.09–1.62	p=0.38
Female	1.1	0.80–1.42		1.2	1.00–1.41	
**Education**						
≤High School	1.3	1.00–1.63	p=0.08	1.3	0.86–1.94	p=0.90
≥College	0.9	0.61–1.27		0.3	0.82–1.94	
**Overweight**						
No	1.0	0.66–1.49	p=0.49	0.9	0.51–1.72	p=0.21
Yes	1.2	0.91–1.49		1.5	1.03–2.06	
[a]*All models controlled for each of the other potential modifiers plus age, physical activity , alcohol consumption, smoking history, saturated fat intake, and fiber*						
[b] *P-value for test of interaction between factor and effect of sugary beverages on outcome*						

© 2019 Collin LJ et al. *JAMA Network Open.*
